# Supplementary material for: Long-term exposure to polychlorinated biphenyl 126 induces liver fibrosis and upregulates miR-155 and miR-34a in C57BL/6 mice
Source: PLoS One. 2024 Aug 12;19(8):e0308334. doi: 10.1371/journal.pone.0308334 (PMC11318903; doi:10.1371/journal.pone.0308334)
Supplement: Attachment — Submitted filename: Response to Reviewers.docx [file pone.0308334.s002.docx]

Dear reviewers,

We greatly appreciate your contribution to improving our article. We have addressed each question point by point and hope that all raised concerns have been adequately answered.

We have worked diligently, returning to the laboratory bench to conduct additional experiments aimed at addressing and working through all the issues raised.

We are available for further clarification if needed.

Sincerely,

Julio Daleprane

**RESPONSE TO REVIWERS**

**Reviewer #1:** The present study "Long-term polychlorinated biphenyl 126 exposure induces liver fibrosis by upregulation of miR-155 and miR-34a in C57BL/6 mice" analyzed some mechanism of fatty liver disease induced by PCB126. Here are some major concerns:

1. microRNA is not epigenetic marker. The authors need to rewrite the manuscript with accurate concepts.

A: We conducted a thorough reading of the entire manuscript and reformulated the sentences.

2. Fibrosis is not an indicator of NASH. If the authors want to prove NASH, infiltration of multiple types of immune cells needs to be experimentally confirmed. Also, the authors need to use experiments to prove hepatocyte death.

A: In response to the reviewer's query regarding our methods to elucidate the development of steatosis into fibrosis, we conducted gene expression assays for markers of different cell types. These included MCP1 for macrophages, CXCL1, and IL-8—key chemokines for neutrophil chemotaxis. Notably, CXCL1 was found to significantly elevate neutrophil infiltration (Figure 2). Additionally, beyond the fibrosis previously demonstrated by picrosirius red staining, we quantified the area occupied by the staining, measured tissue hydroxyproline concentrations, and assessed the expression of pro-fibrotic genes such as TGF-beta1, Smad3, Collagen1, and CXCL9. Cell death was evaluated by assessing Caspase3 expression in hepatic tissue (Figure 3).

Figure 2.

Figure 3.

3. What is the mechanism to regulate miR-155 and miR-34a expression? How is it related to AHR signal?

A: The mechanism by which PCB-126 (polychlorinated biphenyl-126) regulates miR-155 and miR-34a expression is closely tied to its interaction with the aryl hydrocarbon receptor (AhR) signaling pathway. PCB-126 is a potent ligand for AhR, a transcription factor that is activated upon binding with various ligands, including environmental pollutants like dioxins and PCBs. When PCB-126 binds to AHR, it triggers the translocation of the AhR-ligand complex into the nucleus.

Once inside the nucleus, the AhR-ligand complex can bind to specific DNA sequences known as xenobiotic response elements (XREs) located in the promoter regions of various genes, including those encoding for miRNAs like miR-155 and miR-34a. This binding can either upregulate or downregulate the transcription of these miRNAs, depending on the context and the specific interactions at the gene promoters. miR-155 and miR-34a are microRNAs that play crucial roles in various biological processes, including inflammation, cell cycle regulation, and apoptosis. The regulation of these miRNAs by AhR signaling can influence these processes, potentially leading to various biological effects. The alteration of miR-155 and miR-34a expression by PCB-126 through AhR signaling can have significant pathophysiological implications. For example, dysregulation of these miRNAs may contribute to the development of diseases like cancer, where they are known to play roles in regulating apoptosis, proliferation, and the immune response. In summary, PCB-126 regulates miR-155 and miR-34a expression primarily through its interaction with AhR, leading to changes in gene transcription that can have broad biological and health implications. This pathway is a critical aspect of understanding the toxicological effects of PCBs and their impact on cellular and molecular processes.

4. Which cells in the liver express AHR, miR-155 and miR-34a?

A: As described by Yan et al (2019), AhR is expressed in hepatic stellate cells, hepatocytes and Kupffer cells [1]. It has been described that miR-155 is highly expressed in hepatocytes and Kupffer cells, being involved in inflammatory processes that control innate and adaptive immunity in nonalcoholic fatty liver disease, in addition to an important role in the initial hepatic lipid accumulation and modulating lipid metabolism [2]. miR-34a is expressed in hepatocytes and hepatic stellate cells and was associated to induction of hepatocytic apoptosis, regulation of lipid metabolism and stimulation of hepatic stellate cell activation and fibrosis [3].

[1] Yan J, Tung HC, Li S, Niu Y, Garbacz WG, Lu P, Bi Y, Li Y, He J, Xu M, Ren S, Monga SP, Schwabe RF, Yang D, Xie W. Aryl Hydrocarbon Receptor Signaling Prevents Activation of Hepatic Stellate Cells and Liver Fibrogenesis in Mice. Gastroenterology. 2019 Sep;157(3):793-806.e14. doi: 10.1053/j.gastro.2019.05.066. Epub 2019 Jun 3.

[2] Dongiovanni P, Meroni M, Longo M, Fargion S, Fracanzani AL. miRNA Signature in NAFLD: A Turning Point for a Non-Invasive Diagnosis. Int J Mol Sci. 2018 Dec 10;19(12):3966. doi: 10.3390/ijms19123966.

[3] Hochreuter MY, Dall M, Treebak JT, Barrès R. MicroRNAs in non-alcoholic fatty liver disease: Progress and perspectives. Mol Metab. 2022 Nov;65:101581. doi: 10.1016/j.molmet.2022.101581.

5. The authors need to use molecular biology methods to prove whether TNF-alpha and IL-6 are direct targets of miR-155. And whether PPARalpha is a direct target of miR-34a.

A: Unfortunately, it was not possible to perform molecular biology analyzes to prove such associations, but we revised and rewrote the discussion section so that a direct relationship between these markers is not suggested.

“However, these associations have already been well described in previous studies. Tili et al (2007) suggest that miR-155 may act directly or indirectly to increase the rate of translation of TNF- transcripts, possibly through their redistribution across the cytoplasm and/or enhancement of TNF- transcript stability. Furthermore, they showed that miR-155 targets transcripts encoding proteins, such as IKKε (IκB kinase ε), FADD (Fas-associated death domain protein) and Ripk1 (receptor (TNFR superfamily)-interacting serine-threonine kinase 1) whose ultimate function results in the activation of the LPS/TNF- pathway, while it increases TNF- production [1]. In addition, other study described that miR-155 is a master regulator of inflammation, enhancing the translation of TNFα during innate immune responses by Toll-like receptor ligands [2].

Thus it has already been described that the overexpression of miR-155 promoted activation of NF-κB, through mechanisms involving the downregulation of SOCS1, and elevating the production of proinflammatory cytokines, such TNF-α and IL-6 [3, 4]

Concerning to PPARalpha and its relation to mir-34a, previous studies have already reported that the upregulation of miR-34a resulted in the downregulation of hepatic PPARα and SIRT1 that are the direct targets of miR-34a and that silencing of miR-34a restored the expression of SIRT1 and PPARα, resulting in activation of PPARα downstream genes, such as AMPK and Hydroxymethylglutaryl-CoA Reductase (HMGCR) [2, 5].”

[1] Tili E, Michaille JJ, Cimino A, Costinean S, Dumitru CD, Adair B, Fabbri M, Alder H, Liu CG, Calin GA, Croce CM. Modulation of miR-155 and miR-125b levels following lipopolysaccharide/TNF-alpha stimulation and their possible roles in regulating the response to endotoxin shock. J Immunol. 2007 Oct 15;179(8):5082-9. doi: 10.4049/jimmunol.179.8.5082.

[2] Dongiovanni P, Meroni M, Longo M, Fargion S, Fracanzani AL. miRNA Signature in NAFLD: A Turning Point for a Non-Invasive Diagnosis. Int J Mol Sci. 2018 Dec 10;19(12):3966. doi: 10.3390/ijms19123966.

[3] Chen C, Luo F, Liu X, Lu L, Xu H, Yang Q, Xue J, Shi L, Li J, Zhang A, Liu Q. NF-kB-regulated exosomal miR-155 promotes the inflammation associated with arsenite carcinogenesis. Cancer Lett. 2017 Mar 1;388:21-33. doi: 10.1016/j.canlet.2016.11.027.

[4] Tan L, Jiang W, Lu A, Cai H, Kong L. miR-155 Aggravates Liver Ischemia/reperfusion Injury by Suppressing SOCS1 in Mice. Transplant Proc. 2018 Dec;50(10):3831-3839. doi: 10.1016/j.transproceed.2018.08.060.

[5] Ding J, Li M, Wan X, Jin X, Chen S, Yu C, Li Y. Effect of miR-34a in regulating steatosis by targeting PPARα expression in nonalcoholic fatty liver disease. Sci Rep. 2015 Sep 2;5:13729. doi: 10.1038/srep13729.

6. To prove miR-155 and miR-34a participate in the pathogenesis, the authors need to use molecular methods to interfere with the expression of these two microRNAs in the liver and repeat the animal experiment, in comparison to the unrelated interference treatment.

A: Unfortunately, we were unable to perform these experiments, but we revised and rewrote the discussion section to try to minimize this direct association of miRs with the pathogenesis of NASH.

7. Fibrosis is only supported by Sirus Red staining, which is too limited. The authors need to perform hydroxyproline analysis, collagen quantification, fibrosis related gene expression to confirm fibrosis.

A: We performed additional experiments as suggested (Fig 3).

8. What is the mechanism for cholesterol elevation in PCB treated mice?

A: PCB-126 is known to be a potent endocrine disruptor. It can interfere with thyroid and steroid hormone receptors, thereby affecting lipid metabolism in the liver. Changes in hormone levels may result in increased cholesterol synthesis or reduced elimination. Simultaneously, exposure to PCB-126 can alter liver metabolic pathways, increasing cholesterol biosynthesis and/or decreasing its conversion into bile acids. This may be caused by changes in the expression or activity of key enzymes such as HMG-CoA reductase, which is crucial in the cholesterol biosynthetic pathway. Additionally, PCB-126 can induce oxidative stress and inflammation in the liver. Oxidative stress may damage liver cells, while inflammation can trigger a lipid response that includes increased cholesterol synthesis as a defense mechanism. Finally, PCB-126 may affect the function and expression of lipid transporter proteins, such as low-density (LDL) and high-density (HDL) lipoproteins, impacting cholesterol transport and removal from the liver. To improve the quality of the work, we have included the information in the text. [1,2]

1.	Mohammadparast-Tabas P, Arab-Zozani M, Naseri K, Darroudi M, Aramjoo H, Ahmadian H, et al. Polychlorinated biphenyls and thyroid function: a scoping review. Rev Environ Health. 2023. doi:10.1515/reveh-2022-0156

2.	Yang Y, Mei G, Yang L, Luo T, Wu R, Peng S, et al. PCB126 impairs human sperm functions by affecting post-translational modifications and mitochondrial functions. Chemosphere. 2024;346: 140532. doi:10.1016/j.chemosphere.2023.140532

**Reviewer #2:** The authors tested a commonly used pollutant, PCB-126, and observed liver fibrosis and steatosis in mice, as has been shown before. Nevertheless, they also showed increased expression of some miRNAs related to lipid metabolism.
Overall, the results seem concise and nicely presented, and the text is well written, although there are some typos and some mistakes that could need to be revised. Some points of the text are redundant as well, but I’ll bring it along to my revision.

1.Regarding Picrosirius Red, did you make any kind of measurement of the area affected? I would recommend analyzing the area with ImageJ, which is available for free, and several macros could help with the analysis. This could be enriching for the data you show.

A: We performed additional experiments as suggested (Fig 3).

2.The discussion section feels hard to follow. Try to reorganize it to group the results by “themes”. E.g., the Second paragraph (starting in line 245) could come after the third one. Also, this second paragraph gets very redundant with the information that’s about to be presented. Please, reorganize, and check and rewrite what is needed.

A: The discussion section was reviewed and we reorganize and rewrite some paragraphs to make it easier to understand.

3.Statistical-wise, was there any normality test used? It is important to determine if the data was properly analyzed by the Student’s T-test. Also, something that could add to your conclusions, is correlation. Use simple linear correlation to compare the miRNA you measured with other results you observed. E.g., in Figure 3, you add two correlation analysis between IL-6 and TNF-α by the expression of miR-155, and in Figure 4, the same thing with PPAR-α and miR-34a. Other possibilities are evaluating the correlation between the results you got in the stereology of the liver and the expression of these two miR-34a. I understand that, although correlations could be misleading, once you already are exposing the relation in the discussion section that would be justified. Feel free to try other correlations that could work well and present some of them. This could reinforce your results.

A: Dear Reviewer, Thank you for the opportunity to clarify the conduct of our data analyses. To carry out the statistical analyses, we initially performed a normality test on all data, as now described in the methodology section. Subsequent statistical analyses were then performed. We attempted to conduct a correlation test with the studied variables; however, unfortunately, the sample size was too small to establish this relationship. As a result, we changed how we present the results, and no longer claim causality between miRNAs and other inflammatory and fibrotic indicators. We now merely discuss these in light of existing data in the literature.

4.Line 76: …to date, no study…

A: Done.

5.Lines 91 to 97: Information is shown twice. Make the last sentences of the paragraph more complete and you can’t need the subsequent paragraphs.

A: We rewrite the sentence.

6.Line 100: You can use graphical accents on the words that have it originally, as in São Paulo.

A: Done.

7. Line 104: Which drug did you use for euthanasia, pentobarbital or thiopental? Also, use the right symbols {[()]}

A: Sorry for this mistake. The drug used was sodium thiopental and the sentence has already been corrected.

8. Line 122: Reference 20 doesn’t make sense with what is in the text. It’s about chow, not histology.

A:It has been corrected.

9. Lines 170 to 186: Gene names and transcripts are two different things. The gene for TNF-α is Tnfa, and, as this is a rodent gene, the first letter is capitalized and subsequent in lowercase, and italics. For it to be clearer, you can add the proper name of the genes in the parenthesis, before the assay ID. Please, be aware of this and fix it along with the text.
A: We correct this throughout the text.

10. Line 186: Use the proper reference for Livak and Schimittgen.

A: Done.

11. Paragraph starting in line 221: Text is hard to follow. Please, rewrite it to present the data with better flow.

A: We rewrote the paragraph to improve understanding of the data presented.

12. Line 282: Reference of the “other study” should come by the end of the first sentence.

A: The reference was included at the end of the sentence.

13. Line 342: Please, double-check the verbal tense of the verb induce here.

A: Done.

14. Line 346: relation instead of relationship.

A: Done.
