# Supplementary material for: Long-term exposure to polychlorinated biphenyl 126 induces liver fibrosis and upregulates miR-155 and miR-34a in C57BL/6 mice
Source: PLoS One. 2024 Aug 12;19(8):e0308334. doi: 10.1371/journal.pone.0308334 (PMC11318903; doi:10.1371/journal.pone.0308334)
Supplement: Attachment — Submitted filename: Response to Reviewers.docx [file pone.0308334.s003.docx]

Dear Reviewer,

We would like to express our gratitude for the thorough review and valuable feedback on our manuscript. We have carefully considered each of your comments and have made the necessary revisions accordingly. Below, we address each of your concerns in detail:

Major Concern

1. Caspase-3 Expression:

We acknowledge that caspase-3 expression alone cannot definitively indicate apoptosis. In response to your suggestion, we have performed a Western blot analysis to evaluate cleaved caspase-3. Additionally, we have included the total or full-length caspase-3 protein level in our results. The updated data can be found in the revised manuscript (Figure 3I and 3J). We believe this provides a more comprehensive evaluation of apoptosis in our samples.

Minor Concerns:

1. IL-8 Measurement:

We appreciate your observation regarding IL-8. As suggested, we have removed the IL-8 measurement from our manuscript, considering that IL-8 is not typically present in murine models and we have already examined the murine equivalent, CXCL1. The manuscript has been updated accordingly to reflect this change.

2. miR-155 Expression Level:

We have moved the miR-155 expression data from Figure 2 to Figure 4 as requested. This rearrangement aligns better with the overall flow of our results and discussion.

3. Mechanism Linking AHR to miR-155 and miR-34a Regulation:

We have incorporated the information regarding the potential mechanism linking AHR to the regulation of miR-155 and miR-34a into the discussion section of our manuscript. Proper citations have been added to support this information. We believe this addition strengthens the discussion and provides a clearer understanding of the mechanisms involved.

We sincerely thank you again for your constructive feedback, which has significantly improved the quality of our manuscript. We hope that the revised version meets your expectations and addresses all your concerns satisfactorily.

Thank you for your time and consideration.

Best regards,

Julio Daleprane
